# Plant-Derived Spinacetin Mitigates Cyclophosphamide-Induced Hemorrhagic Cystitis in Rats

**DOI:** 10.3390/ijms27073056

**Published:** 2026-03-27

**Authors:** Jan Wróbel, Łukasz Zapała, Grzegorz Niemczyk, Anna Bogaczyk, Tomasz Kluz, Artur Wdowiak, Aleksandra Misiek, Iwona Bojar, Ewa Poleszak, Marcin Misiek, Kinga Gaweł, Andrzej Wróbel

**Affiliations:** 1 Medical Faculty, Medical University of Lublin, 20-093 Lublin, Poland; 2 Clinic of General, Oncological and Functional Urology, Medical University of Warsaw, Lindleya 4 Lindleya 4 St., 02-005 Warsaw, Poland; grzegorz.niemczyk@wum.edu.pl; 3 Department of Gynecology, Gynecology Oncology and Obstetrics, Faculty of Medicine, University of Rzeszow, Tadeusza Rejtana 16 c St., 35-959 Rzeszow, Poland; annabogaczyk@interia.pl (A.B.); jtkluz@interia.pl (T.K.); 4 Obstetrics and Gynecology, Faculty of Health Sciences, Medical University of Lublin, Staszica 4-6 St., 20-081 Lublin, Poland; wdowiakartur@gmail.com; 5 Institute of Medical Sciences, Jan Kochanowski University, 25-369 Kielce, Poland; olamisiek@icloud.com; 6 Department of Women’s Health, Institute of Rural Health in Lublin, Jaczewskiego 2 St., 20-090 Lublin, Poland; iwonabojar75@gmail.com; 7 Department of Applied and Social Pharmacy, Laboratory of Preclinical Testing, Medical University of Lublin, Chodźki 1 St., 20-093 Lublin, Poland; ewapoleszak@umlub.pl; 8 Department of Gynecologic Oncology, Holy Cross Cancer Center, 25-377 Kielce, Poland; marcin.misiek@onkol.kielce.pl; 9 Faculty of Medicine, Collegium Medicum, Jan Kochanowski University in Kielce, 25-369 Kielce, Poland; 10 Department of Pharmacology, Poznan University of Medical Sciences, Rokietnicka 3 Str., 60-806 Poznan, Poland; kgawel.farmakologia@gmail.com; 11 Second Department of Gynecology, Medical University of Lublin, Jaczewskiego 8 St., 20-090 Lublin, Poland; wrobelandrzej@yahoo.com

**Keywords:** cyclophosphamide, hemorrhagic cystitis, spinacetin

## Abstract

The purpose of our study was to assess if spinacetin (SPC), a flavonoid found in spinach, can alleviate the cyclophosphamide (CYP)-induced changes in cystometric and inflammatory parameters indicative of the development of hemorrhagic cystitis. The animal experiments were conducted in female Wistar rats. The cohort of 60 animals was grouped as follows: I—control, II—CYP group, III—SPC group, and IV—CYP + SPC group. The cystometry and biochemical analyses were performed after a fortnight of SPC administration. SPC was found to restore normal cystometric parameters in CYP-induced cystitis and, similarly, it normalized c-Fos expression changes in the central micturition regions. SPC further prevented a massive increase in the bladder wall thickness/permeability due to exposition to CYP administration. CYP instillation resulted in the elevation of biomarkers found in urine (brain-derived neurotrophic factor, BDNF, and nerve growth factor, NGF), and in the bladder detrusor muscle (Rho kinase and vesicular acetylcholine transporter, VAChT), which were successfully restored after administration of SPC. As for the biomarkers in the bladder urothelium, the CYP-induced increases in TNF-α, IL-1β, IL-6, calcitonin gene-related peptide (CGRP), malondialdehyde, 3-nitrotyrosine, insulin-like growth factor-binding protein 3 (IGFBP-3), occludin, organic cation transporter 3 (OCT-3), orosomucoid-1 (ORM1), pituitary adenylate cyclase receptor 1 (PAC1), synaptosomal-associated protein 23 (SNAP23), SNAP25, and synaptic vesicle glycoprotein (SV2A) levels were attenuated by SPC. Finally, CYP administration resulted in a decrease in the heparin-binding EGF-like growth factor (HB-EGF), hemopexin (HPX), T-H protein, and tight junction protein (Z01), and we noted the successful restoration of all these changes in concentrations after application of SPC. In summary, SPC robustly mitigated cyclophosphamide (CYP)-induced cystometric dysfunction and biochemical alterations characteristic of iatrogenic hemorrhagic cystitis. These findings position SPC as a compelling therapeutic candidate and warrant further translational investigation for the management of CYP-induced bladder injury.

## 1. Introduction

Cyclophosphamide (CYP) remains the current treatment of several oncological diseases such as breast, lymphoid and pediatric malignancies [1]. Due to its potent immunosuppressive properties, it also commonly serves as a medical agent in blood and marrow transplantation indications [1]. Despite its wide implementation, one should be aware of possible toxic effects, including bone marrow suppression and cardiac and gonadal toxicity, with the risk directly connected with cumulative dose [1,2].

The other tremendous possible side effect of CYP is well-described hemorrhagic cystitis. Although known since the late 1970s, it is still continuously perceived as a debilitating condition with hardly any effective medical modalities to treat it [2]. Being the most common form of CYP-induced bladder toxicity [3], the timing of its occurrence varies in different individuals, with the early phase of cystitis originally attributed to acrolein [1]. On the other hand, the condition is found in over ¼ of cases with late onset, in whom a high-dose treatment is implemented [4]. CYP-induced hemorrhagic cystitis is mainly manifested by gross hematuria and irritative voiding symptoms during treatment. However, even many years after completion of treatment, a substantial number of patients have persisting irritative voiding symptoms or recurrence of gross hematuria [3]. It has also been proven that CYP may induce nerve injury, resulting in bladder pain and an overactive bladder in a rat model by the upregulation of Nlrp6 and Casp11 [5]. CYP induces hemorrhagic cystitis by renal excretion of acrolein, its hepatic metabolite with urotoxic properties [1]. Miodosky et al. reported protective effects of hyaluronate via various actions, including inhibition of immune complexes, leukocyte migration, regulation of fibroblast and endothelial cell proliferation, and, finally, enhancement of connective tissue healing [6]. What is more, acrolein urotoxicity increases the risk of bladder cancer from 9-fold to 40-fold [1,3]. In a recent systematic review, Chou et al. revealed that these tumors arising in the bladder had a propensity for a younger age and a more advanced stage at diagnosis, and variant histology [7]. Thus, there is a strong demand for drugs that can reduce the toxicity of CYP metabolites.

In our previous papers, we have noted that natural herbal derivatives, due to their impressive alleviative properties, may be an option in the future treatment of this condition, as we presented in a rat model [8,9]. Among other possible candidates, spinacetin (SPC) was proposed, which is a flavonoid found in some plants, notably spinach (*Spinacia oleracea*), which is where the name comes from [10,11]. It is thought to possess a broad range of interesting biological properties, including anti-inflammatory, cardio- and nephroprotective, anti-lipidemic and antioxidative ones [1,11,12]. Of note, several authors have clearly demonstrated the beneficial effects of spinacetin; however, spinach contains several other constituents that may exhibit similar biological activities, e.g., ternatin [13]. On the other hand, SPC, being the important ingredient in the spinach plant, has been connected to spinach’s lipid-lowering effects and cardiovascular protection [1,14]. Thus, Liu and Zhao determined that SPC could protect against doxorubicin-induced cardiotoxicity both in vitro and in vivo by initiating protective autophagy through SIRT3/AMPK/mTOR (sirtuin 3/AMP-activated protein kinase/mammalian target of rapamycin) [14]. On the other hand, a multidimensional impact of SPC administration was noted by Bibi et al. [15]. The authors reported significant protection of hepatic tissues against cadmium-exacerbated ischemic/reperfusion injury in rats through the regulation of mitochondrial biogenesis, oxidative stress, inflammation, and apoptosis [15]. Yet, little is known about its potential effects on lower urinary tracts, although some possible similar actions in other organs have been observed, e.g., antinephrolithiatic effects in in vitro assays (urease inhibition assay) or spasmolytic effects in an in vivo rat model [16].

Thus, the current investigation was carried out to establish if any protective determinants of SPC exist in CYP-induced hemorrhagic cystitis in a rat model. We focused on the experiments to ascertain if SPC ameliorates the CYP-induced changes in several cystometric and inflammatory parameters, indicating the development of bladder inflammation and bladder overactivity.

## 2. Results

### 2.1. The Effects of SPC on Cyclophosphamide (CYP)-Induced Changes in Cystometric Parameters

As previously described [5], a cyclophosphamide injection led to a significant increase in the following parameters in the filling phase of conscious cytometry: the basal pressure (BP), threshold pressure (TP), detrusor overactivity index (DOI), non-voiding contraction frequency (FNVC), non-voiding contraction amplitude (ANVC), volume threshold to elicit NVC (VTNC), and bladder compliance (BC) (Table 1). These findings indicative of bladder overactivity were followed by the effects also observed in the voiding phase, that is, the decrease in the intercontraction interval (ICI) and voided volume (VV), and the increase in the area under the pressure curve (AUC), with no influence found on the post-void residual (PVR). Interestingly, we observed that, not only did SPC administration fail to result in similar effects when compared to the CYP group, but this agent also attenuated the influence of CYP on all the above-mentioned parameters in both the storage and voiding phases.

### 2.2. Effects of SPC on Cyclophosphamide (CYP)-Induced Changes in Urothelium Thickness and in Vesical Vascular Permeability in Evans Blue Dye Assessment of Bladder Edema

**Figure 1:**
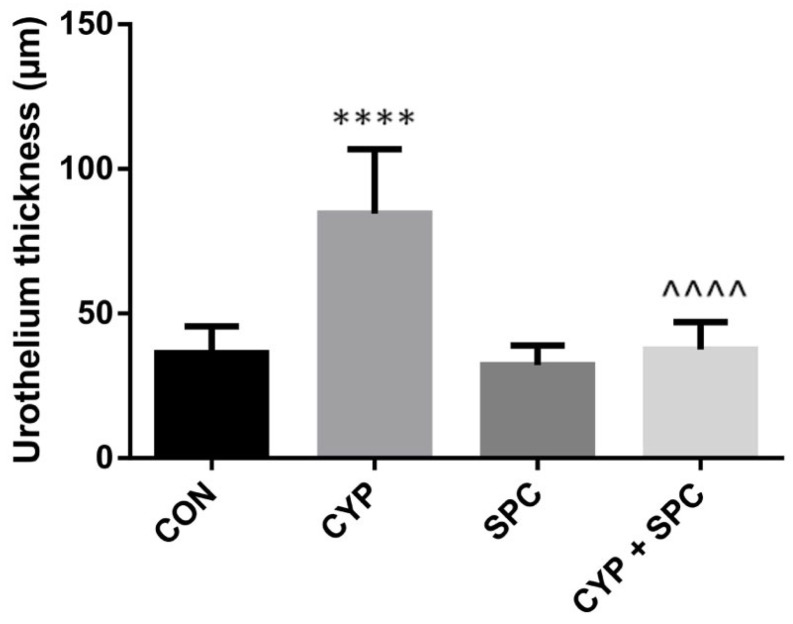
The effects of spinacetin (SPC, 20 mg/kg/day) administered for 14 consecutive days on cyclophosphamide (CYP)-induced changes in the urothelium thickness. Values are expressed as the mean ± SD. Abbreviations: control group (CON), CYP group (CYP), SPC-only group (SPC) and CYP group treated with SPC (CYP + SPC). **** or ^^^^ *p* < 0.0001. * significantly different from the control group. ^ significantly different from the CYP group.

**Figure 2:**
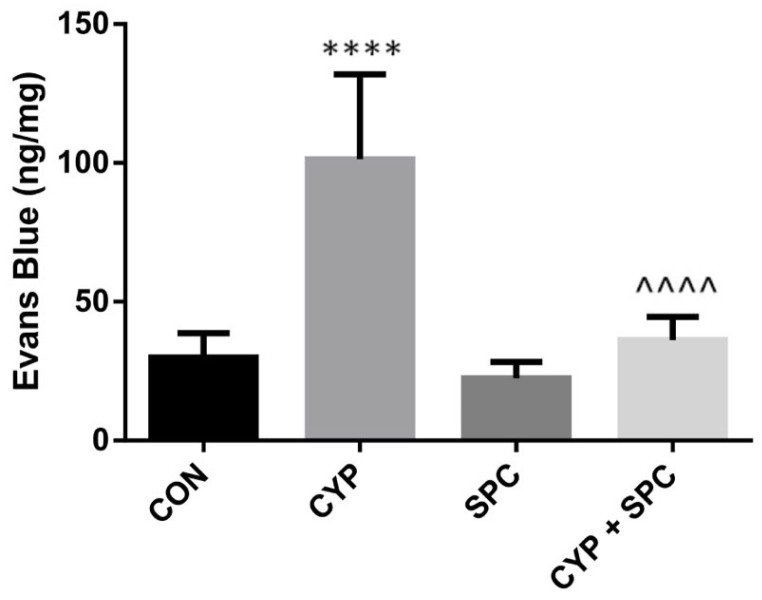
The effects of spinacetin (SPC, 20 mg/kg/day) administered for 14 consecutive days on cyclophosphamide (CYP)-induced changes in bladder edema. Abbreviations: control group (CON), CYP group (CYP), SPC-only group (SPC) and CYP group treated with SPC (CYP + SPC). Values are expressed as the mean ± SD. **** or ^^^^ *p* < 0.0001. * significantly different from the control group. ^ significantly different from the CYP group.

We recently presented that our model of CYP-induced cystitis allows the effects of novel agents on the mucosal inflammatory response to be estimated, as indicated by macroscopic changes within the urinary bladder [12]. Hence, in the current study, we found that SPC prevented a massive increase in the bladder wall thickness due to exposition to CYP administration (Figure 1). Furthermore, the bladder edema measured as one of the CYP-induced changes was attenuated by the SPC treatment (Figure 2).

### 2.3. The Effects of SPC on Cyclophosphamide (CYP)-Induced Changes on the Expression Levels of c-Fos in Central Micturition Areas

**Figure 3:**
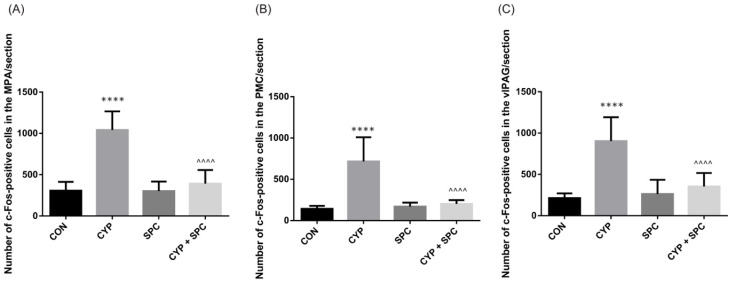
The effects of spinacetin (SPC, 20 mg/kg/day) on c-Fos expressions in the neuronal voiding centers—(**A**) medial preoptic nucleus (MPA), (**B**) pontine micturition center (PMC), and (**C**) ventrolateral periaqueductal gray (vlPAG)—after the induction of CYP-induced changes. The number of c-Fos-positive cells in each group is shown: control group (CON), CYP group (CYP), SPC-only group (SPC) and CYP group treated with SPC (CYP + SPC). Values are expressed as the mean ± SD. **** or ^^^^ *p* < 0.0001. * significantly different from the control group. ^ significantly different from the CYP group.

Then, we focused on the changes in the c-Fos expression after CYP administration in all the analyzed central micturition compartments, when collated to the control group, and noted substantial increases in all the centers (Figure 3). Then, we found that SPC demonstrated a potential to significantly lower the expression of c-Fos after CYP exposure (CYP-plus-SPC group compared to CYP group) in all three studied micturition areas (MPA, PMC, and vlPAG).

### 2.4. The Effects of SPC on Cyclophosphamide (CYP)-Induced Changes in Biochemical Analyses of Biomarkers in Urine

**Figure 4:**
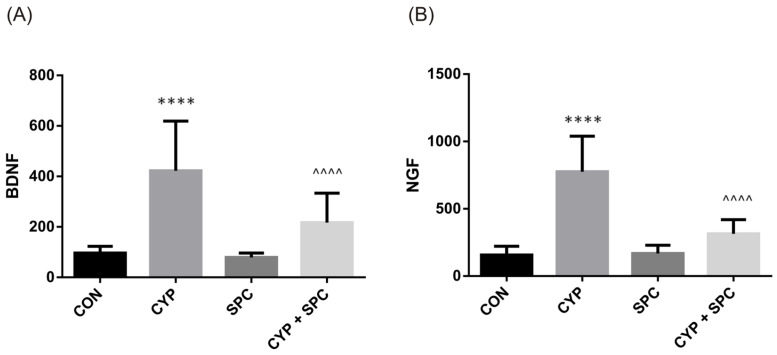
The influence of the 14-day administration of SPC on biomarkers in urine—(**A**) BDNF and (**B**) NGF—in rats subjected to cyclophosphamide (CYP). Values are expressed as the mean ± SD. **** *p* < 0.001 versus saline, ^^^^ *p* < 0.0001 versus CYP (n = 15 rats per group). CON, control.

**Figure 5:**
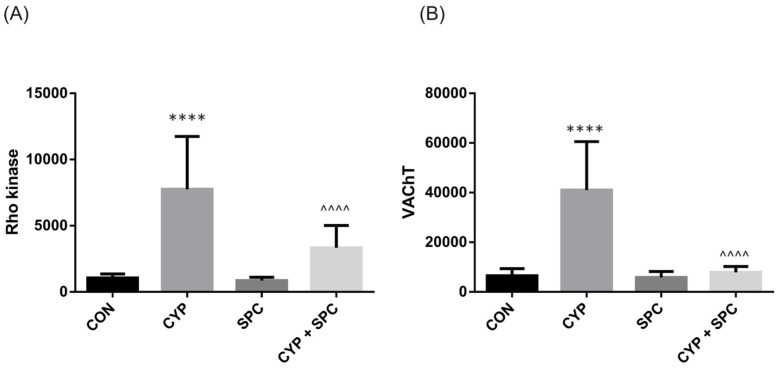
The influence of the 14-day administration of SPC on biomarkers in the bladder detrusor muscle—(**A**) Rho kinase and (**B**) VAChT—in rats subjected to cyclophosphamide (CYP). Values are expressed as the mean ± SD. **** *p* < 0.001 versus saline, ^^^^ *p* < 0.0001 versus CYP (n = 15 rats per group). CON, control.

**Figure 6:**
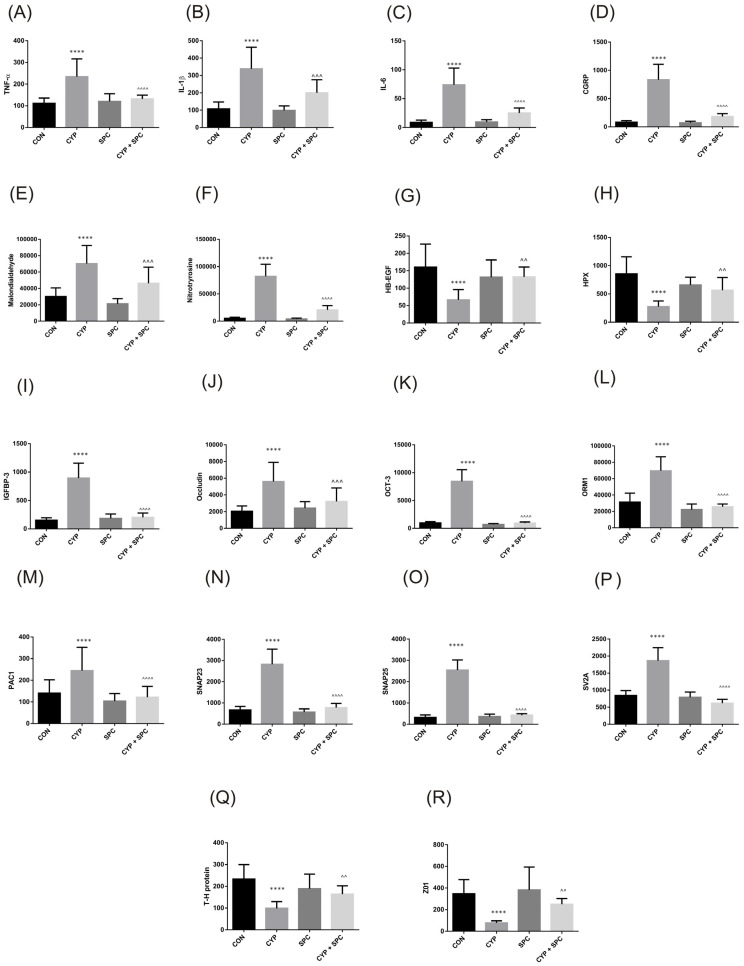
The influence of the 14-day administration of SPC on biomarkers in the bladder urothelium—(**A**) TNF-α, (**B**) IL-1β, (**C**) IL-6, (**D**) CGRP, (**E**) malondialdehyde, (**F**) 3-nitrotyrosine, (**G**) HB-EGF, (**H**) HPX, (**I**) IGFBP-3, (**J**) occludin, (**K**) OCT3, (**L**) ORM1, (**M**) PAC1, (**N**) SNAP23, (**O**) SNAP25, (**P**) SV2A, (**Q**) T-H, and (**R**) Z01—in rats subjected to cyclophosphamide (CYP). Values are expressed as the mean ± SD. **** *p* < 0.001 versus saline, ^^ *p* < 0.01, ^^^ *p* < 0.001, ^^^^ *p* < 0.0001 versus CYP (n = 15 rats per group). CON, control.

No significant changes in the levels of the analyzed biomarkers after administration of SPC only were found, when collated with the saline-treated group (Figure 4, Figure 5 and Figure 6). CYP instillation resulted in the elevation of biomarkers found in urine, i.e., BDNF and NGF (please refer to Figure 4), while their levels were successfully restored after administration of SPC (CYP + SPC combination group, Figure 4).

### 2.5. The Effects of SPC on Cyclophosphamide (CYP)-Induced Changes in Biochemical Analyses of Biomarkers in the Detrusor

Similarly to the urine biomarkers, CYP administration led to an increase in both Rho kinase and VAChT measured in the bladder detrusor muscle (Figure 5). The administration of SPC ameliorated those findings.

### 2.6. The Effects of SPC on Cyclophosphamide (CYP)-Induced Changes in Biochemical Analyses of Biomarkers in Bladder Urothelium

Then, we focused on the compartment of the bladder urothelium for the effects of CYP administration and assessed the possible protective effects of SPC in this setting. We observed that a CYP injection resulted in an increase in TNF-α, IL-1β, IL-6, CGRP, malondialdehyde, 3-nitrotyrosine, IGFBP-3, occludin, OCT-3, ORM1, PAC1, SNAP23, SNAP25, and SV2A (Figure 6). On the contrary, CYP administration led to a significant decrease in the following biomarkers assessed in the urothelium: HB-EGF, HPX, the T-H protein, and Z01. We further observed the successful restoration of all these changes in concentrations after application of SPC (CYP + SPC combination group).

## 3. Discussion

Here, for the first time, we present the impressive potential of SPC in reversing some of the mechanisms of side effects of CYP in a rat model of hemorrhagic cystitis. Importantly, cyclophosphamide-induced hemorrhagic cystitis represents a well-established experimental model that recapitulates key features of a secondary overactive bladder associated with urothelial injury, inflammation, and afferent sensitization. Previous studies using this model have demonstrated that CYP-induced detrusor overactivity is accompanied not only by peripheral inflammatory cascades, but also by profound alterations in sensory signaling pathways, including enhanced neurotrophin release, upregulation of excitatory neurotransmission, and increased activity within central micturition centers [8,9]. Such changes result in a lowered threshold for bladder afferent firing and exaggerated voiding reflexes, which are reflected cystometrically by reduced intercontraction intervals and increased non-voiding contractions. Therefore, the observed normalization of both filling- and voiding-phase parameters following SPC administration strongly suggests that SPC’s protective effects extend beyond structural preservation of the urothelium and encompass functional modulation of inflammatory–neurogenic mechanisms driving CYP-induced OAB.

Acrolein, a cytotoxic metabolite of CYP, causes bladder structural alterations as edema, necrosis, ulceration, hemorrhage, leukocyte infiltration and neovascularization [3]. Similarly in our study, we found that CYP generated edema of the urothelium and an increased bladder thickness. Importantly, these adverse structural changes were significantly attenuated by SPC, which proves its protective effect. It could be of the utmost importance in the context of CYP-induced bladder remodeling, which eventually could lead to bladder fibrosis and contraction [1,3].

Patients treated with CYP present with irritative voiding symptoms such as frequency, dysuria, urgency, incontinence and nocturia [3]. In the literature, there is a lack of studies that have urodynamically evaluated patients with CYP-induced cystitis, and it is impossible to directly replicate voiding symptoms in animals [17]. Those symptoms, as in other studies, can be indirectly confirmed by cystometric findings [18], especially, as in our study, by increased DOI and FNVC, as well as decreased ICI and VV, as a result of SPC administration.

Functional brain imaging studies in humans have identified brain micturition centers, for which the activity in patients with overactive bladder syndrome is enhanced [19,20]. In turn, in animals, activity of micturition centers may be assessed by analyzing the c-Fos expression, which is the marker of neuronal activity [21]. After CYP administration, c-Fos expression in brain micturition centers (i.e., MPA, PMC and vlPAG) was increased, which corresponds well with the observed functional bladder dysfunction. In such a way, SPC exhibited a protective effect by reversing the functional changes induced by CYP.

TNF-α, IL-1β and IL-6 are proinflammatory cytokines that are found in patients with interstitial cystitis [22]. In our study, all of them were significantly increased in the bladder urothelium after administration of CYP, which is indicative of robust inflammation. This fact was also confirmed by upregulation of ORM1, which is an acute-phase protein [23]. On the other hand, malondialdehyde and 3-nitrotyrosine are both markers of oxidative stress in response to inflammation [24,25]. Excessive oxidative stress leads to cell damage and cell death, as well as contributes to voiding dysfunction manifested by detrusor overactivity [25]. In our present paper, we reveal that SPC exhibited strong anti-inflammatory and antioxidant effects, counteracting CYP action by lowering the levels of all these biomarkers.

We further observed in the current paper that HPX expression was reduced after CYP administration. Interestingly, it has also been proven that HPX is downregulated in patients with bladder pain syndrome [26]. We may hypothesize that hematuria and hemorrhage observed in CYP cystitis may lead to hemoglobin breakdown and heme release with subsequent HPX depletion.

NGF and BDNF have been extensively studied in bladder dysfunction research due to their role in modulating neuronal function in micturition [27]. Urine NGF is thought to be elevated in patients with overactive bladder syndrome, and its level is correlated with symptoms severity [27,28]. Successful treatment with antimuscarinic drugs and botulinum toxin A significantly decreases level of urinary NGF [28]. In this context, by reducing level of urinary NGF, SPC may also become effective in reducing irritative symptoms. NGF is also an established inductor of PAC1 expression [29]. PAC1 is a receptor for pituitary adenylate cyclase-activating polypeptide (PACAP) [29]. PACAP and PAC1 are upregulated in the bladders of rats with CYP-induced cystitis, as we described above, as well as in the bladders of patients with bladder pain syndrome [30]. A blockade of PAC1 in rats with CYP-induced cystitis may improve BC, reduce the voiding frequency and decrease FNVC [29], making this another interesting mechanism that the action of SPC is involved in.

Rho kinase is an important regulator of smooth muscle tone and contractions in healthy bladders [31]. However, in a pathological state, Rho kinase expression is increased and contributes to detrusor overactivity, as we reported previously [32]. VAChT expression may be directly stimulated by NGF and BDNF [33]. Furthermore, it is positively correlated with ACh release and may, therefore, enhance neuronal cholinergic transmission [34]. In our study, CYP administration increased expression of both Rho kinase and VAChT, which partially explains the bladder overactivity found in cystometry. On the other hand, Rho kinase inhibition led to a significant improvement in bladder function, as we described elsewhere [32]. Interestingly, in our current study, decreased expression of Rho kinase and VAChT caused by SPC administration led to the improvement of bladder function.

Urothelium is a non-neuronal source of ACh [35]. After a stimulus, Ach is released from the urothelium via the OCT3 pathway [35]. In our study, CYP increased expression of OCT3 in the urothelium, which strongly suggests the elevation of ACh release. CGRP is released from peripheral axons because of a noxious stimulus [36]. It has a putative role in peripheral sensitization and, via modulation of the bladder reflex, its action results in bladder overactivity, as noted in our study [26,37].

SNAP-25 and SNAP-23 belong to the SNARE complex, which is essential for exocytosis of numerous neurotransmitters and molecules [38]. SNAP-25 has a well-established function in ACh release in efferent neurons, while in the urothelium, both SNAP-25 and SNAP-23 may release other transmitters, such as ATP [35]. SV2A is responsible for neurosecretion, and it also enables the botulinum toxin to be transferred to the cytoplasm where it can exert its action [39]. In line with our study, SV2A expression was increased in patients with detrusor overactivity and painful bladder syndrome [39].

Both occludin and ZO-1 constitute a tight junction in apical cells of the urothelium and regulate paracellular transport [40]. According to the reports that CYP-induced cystitis caused bladder ulceration [41] and led to increased permeability of the urothelial barrier [42], we observed decreased expression of ZO-1. Surprisingly, we also observed an increased expression of occludine, which may suggest that the tight junction structure is substantially altered.

Beyond disruption of the urothelial structure, its regenerative capacity may also be reduced by CYP. Here, we observed that HB-EGF expression, a factor contributing to urothelial proliferation and regeneration after a mucosal injury [43], was downregulated due to CYP-induced changes and restored via SPC action. Similarly, in patients with interstitial cystitis, a low level of HB-EGF in urine seems to be suggestive of the disease [44].

Finally, IGFBP-3 is a putative protective agent against bladder hypertrophy and remodeling induced by insulin growth factor [45]. Interestingly, in our study, IGFBP-3 was upregulated after CYP administration, and likewise in patients with interstitial cystitis, as reported by Erickson et al. [46]. This may indicate the existence of an innate defense mechanism acting against harmful stimuli.

From a clinical perspective, the observed effects of SPC in the cyclophosphamide-induced model of overactive bladder suggest several important translational implications. Firstly, SPC targets fundamental pathophysiological mechanisms underlying CYP-induced OAB, including urothelial damage, inflammatory activation, oxidative stress, and afferent hypersensitivity [47,48]. This multimodal mode of action is particularly relevant in iatrogenic bladder dysfunction, where conventional antimuscarinic therapy often provides limited benefit due to its inability to address inflammation-driven sensory dysregulation. Secondly, by restoring the urothelial integrity and tight junction signaling while simultaneously attenuating neurogenic inflammation and abnormal neurotransmitter release, SPC may reduce urgency and frequency symptoms without directly suppressing the physiological detrusor contractility, potentially translating into a favorable tolerability profile. Thirdly, given that CYP-induced cystitis frequently complicates oncological therapy and may necessitate a dose reduction or discontinuation of chemotherapy, SPC could represent an adjunctive strategy aimed at preserving bladder function and improving quality of life without interfering with the antineoplastic efficacy. Finally, the efficacy of SPC in normalizing cystometric parameters supports its potential role as a disease-modifying rather than solely symptomatic agent in secondary OAB associated with chemotherapy-induced bladder injury. Collectively, these features justify further clinical exploration of SPC as a novel, mechanism-based therapeutic option for patients suffering from CYP-induced OAB.

There are some limitations of our study worth pinpointing. Our study group comprised only female animals, and this necessitates further experiments to be performed on male rats. The effects of SPC should not be extrapolated directly from our in vitro and in vivo tests to human populations; this calls for clinical trials. As for the dosages and treatment schedule, these were determined based on preliminary unpublished data, as no strict analyses could be taken from the available literature.

## 4. Materials and Methods

### 4.1. Study Objective

The primary objective of this study was to evaluate whether spinacetin could counteract cyclophosphamide (CYP)-induced alterations in cystometric and biochemical parameters associated with the development of bladder overactivity and inflammation. The study further aimed to determine whether spinacetin could represent a viable therapeutic strategy for the management of CYP-induced hemorrhagic cystitis. All experimental procedures were reviewed and approved on 10 October 2022 by Local Ethical Committee No. 46 (the approval number is 4326/LKB/323/2022) and were performed in accordance with European legislation governing animal experimentation, including the ARRIVE guidelines and the EU Directive 2010/63/EU.

### 4.2. Animals

A total of sixty adult female Wistar rats, initially weighing 200–225 g, were used in this study. The animals were individually housed in metabolic cages (model 3700M071, Tecniplast, West Chester, PA, USA) under standardized environmental conditions: the temperature maintained at 22–23 °C, a natural light/dark cycle, and a relative humidity of approximately 45–55%. Food and water were provided ad libitum throughout the experimental period.

The animals were randomly allocated into four experimental groups (n = 15 per group):

- Control group (CON): this group received a single administration of vehicle I and vehicle II for 14 consecutive days.
- CYP group: this group received a single intraperitoneal injection of cyclophosphamide (200 mg/kg) and vehicle II for 14 days.
- SPC group: this group was treated with vehicle I and spinacetin (20 mg/kg/day) for 14 days.
- CYP + SPC group: this group received cyclophosphamide (200 mg/kg) combined with spinacetin (20 mg/kg/day) for 14 days.

### 4.3. Drugs

The following pharmacological agents were employed in the present study:

- Cyclophosphamide (CYP)—Endoxan, Baxter Deutschland GmbH, Unterschleißheim, Germany.
- Spinacetin (SPC)—3,5,7-Trihydroxy-2-(4-hydroxy-3-methoxyphenyl)-6-methoxy-4H-1-benzopyran-4-one (CAS No. 3153-83-1; Cat. No. HY-N10182; MedChemExpress, Monmouth Junction, NJ, USA).

Cyclophosphamide was dissolved in physiological saline (0.9% NaCl) and administered intraperitoneally (i.p.) as a single dose of 200 mg/kg. Treatment with spinacetin (20 mg/kg/day) was initiated thereafter and continued for 14 consecutive days. Spinacetin was prepared in a 1% dimethyl sulfoxide (DMSO) solution and administered by oral gavage. Control animals received volume-matched vehicle treatments: vehicle I (intraperitoneal injection of physiological saline, 0.9% NaCl) and/or vehicle II (1% DMSO solution administered orally). Both the drug dosages and treatment regimens were selected based on previously published data [47,49] and earlier experiments conducted in our laboratory [50], and were further validated in preliminary studies. Cystometric evaluations, as well as assessments of bladder edema, the urothelial thickness, and biochemical parameters, were carried out three days after the final administration of spinacetin.

### 4.4. Surgical Procedures

All surgical procedures were conducted in accordance with previously established protocols [47,49]. General anesthesia was induced and sustained with inhaled isoflurane administered in an oxygen-enriched mixture. A sufficient anesthetic depth was verified before initiation of the experimental procedure and continuously evaluated thereafter through the absence of reflexive responses. The depth of anesthesia was confirmed by the lack of spontaneous motor activity and the absence of a withdrawal response following a toe pinch stimulation. Throughout the experiment, the animals were closely monitored to maintain stable physiological parameters. The animals were positioned supine on a thermostatically controlled heating pad (maintained at 37 °C) to ensure normothermia. After shaving and disinfecting the abdominal region, a vertical midline incision (approximately 10 mm) was made through the abdominal wall. The urinary bladder was carefully exposed and separated from surrounding tissues. A double-lumen polyethylene catheter (inner diameter = 0.28 mm; outer diameter = 0.61 mm; BD, Franklin Lakes, NJ, USA) filled with physiological saline and equipped with a cuff at the distal end was inserted into the bladder apex through a small incision. The catheter was secured with a 6-0 Vicryl suture. To prevent postoperative adhesions, 0.85 mL of Healon (Pharmacia AB, Uppsala, Sweden) was gently applied around the bladder. The abdominal wall was then closed in multiple layers, with each anatomical layer sutured using 4-0 catgut thread. The external ends of the catheters were sealed with silk ligatures. Postoperatively, each rat received a subcutaneous injection of cefazolin sodium hydrate (100 mg; Biofazolin, Sandoz, Holzkirchen, Bavaria, Germany) to minimize the risk of a urinary tract infection.

### 4.5. Conscious Cystometry

Cystometric assessments in conscious animals were carried out on day 17 following the surgical intervention, corresponding to three days after the final administration of spinacetin, as previously described [8,9]. The indwelling bladder catheter was connected via a three-way stopcock to both a pressure transducer (FT03, Grass Instruments, Quincy, MA, USA) positioned at the level of the urinary bladder and a microinfusion pump (CMA 100, Microject, Solna, Sweden). This setup allowed for simultaneous measurement of the intravesical pressure and controlled infusion of physiological saline into the bladder. Cystometric recordings were performed in awake, freely moving rats during continuous bladder filling with physiological saline at room temperature (22 °C). The infusion rate was maintained at 0.05 mL/min (equivalent to 3 mL/h), which was determined in preliminary trials to produce cystometric patterns comparable to those observed in normal rat micturition reflexes. Infusion rates exceeding this range (≥0.1 mL/min) typically led to an increased bladder capacity or provoked reflex detrusor contractions. The pressure signal from the transducer was amplified and digitized using the Polyview data acquisition system (Grass Instruments). Voided volumes were quantified with a fluid collector connected to a force-displacement transducer (FT03C, Grass Instruments). Both transducers were linked to a polygraph (Model 7DAG, Grass Instruments) for synchronous recording of pressure and volume data. Cystometric traces and micturition volumes were continuously monitored and recorded on a Grass Model 7E polygraph, followed by graphical evaluation of the recorded data. Data acquisition was conducted at a sampling rate of 10 Hz. For each animal, the results were averaged from five consecutive and reproducible micturition cycles obtained after the establishment of regular voiding activity. Group means were subsequently calculated by averaging the data from all animals within each experimental condition. All cystometric procedures and analyses were performed by an experimenter blinded to the treatment allocation.

### 4.6. Biochemical Analyses

Levels of the following biomarkers were determined in the bladder urothelium: calcitonin gene-related peptide (CGRP; Biomatik, Cambridge, ON, Canada, CN EKU02858), tumor necrosis factor a (TNF-α; LifeSpan BioSciences, Seattle, WA, USA, CN LS-F5193), interleukin 1-β (IL-1β; Cloud-Clone, Katy, TX, USA, CN SEA563Ra), interleukin 6 (IL-6; LifeSpan BioSciences, Seattle, WA, USA; CN LS-F25921-1), organic cation transporter 3 (OCT3; antibodies-online, Aachen, North Rhine-Westphalia, Germany, CN ABIN6227163), Tamm–Horsfall protein (T-H protein, uromodulin; Antibodies-online, Aachen, North Rhine-Westphalia, Germany; CN ABIN855058), heparin-binding epidermal growth factor-like growth factor (HB-EGF; Biomatik, Cambridge, ON, Canada; CN EKU04742), 3-nitrotyrosine (NIT; LifeSpan BioSciences, Seattle, WA, USA; CN LS-F40120-1), malondialdehyde (MAL; Biomatik, CN EKF57996), tight junction protein 1 (ZO1; CUSABIO, Wuhan, China, CSB-E17287r), pituitary adenylate cyclase receptor 1 (PAC1; LifeSpan BioSciences, LS-F17843), synaptosome-associated protein 25 (SNAP-25; Biomatik, EKF58391), synaptosome-associated protein 23 (SNAP-23; MyBioSource, San Diego, CA, USA, MBS9317604), rat synaptic vesicle glycoprotein 2A (SVG2A; MyBioSource, San Diego, CA, USA; MBS9348576), orosomucoid-1 (ORM1; MyBioSource), hemopexin (HPX; MyBioSource), occludin (OCC, MyBioSource) and insulin-like growth factor-binding protein 3 (IGFBP-3; MyBioSource). The levels of the vesicular acetylcholine transporter (VAChT; LifeSpan BioSciences, CN LS-F12924-1) and Rho kinase (ROCK1; LifeSpan BioSciences, LS-F32208) were marked in the bladder detrusor muscle. Additionally, urine samples were collected for an estimation of the brain-derived neurotrophic factor (BDNF; PROMEGA, Madison, WI, USA, CN G7610) and nerve growth factor (NGF; LifeSpan BioSciences, CN LS-F25946-1). All measurements were carried out according to the manufacturers’ instructions as previously described [32]. Each sample was measured in duplicate. The results are presented in pg/mL.

### 4.7. Assessment of Bladder Edema

Bladder edema was quantified by evaluating the vesical vascular permeability using the Evans Blue dye extravasation method, as previously described [9,51]. Briefly, Evans Blue was administered intravenously at a dose of 50 mg/kg via a catheter inserted into the right femoral vein. Thirty minutes after dye injection, the animals were euthanized, and their urinary bladders were excised, weighed, and longitudinally bisected. Each specimen was then immersed in 1 mL of formamide and incubated at 56 °C for 24 h to extract the dye. The optical density of the resulting supernatant was measured at 620 nm using a spectrophotometric analysis, and the dye concentration was determined based on a standard calibration curve. The degree of vascular permeability was expressed as nanograms of Evans Blue per milligram of bladder tissue.

### 4.8. Assessment of Urothelium Thickness

The urothelial thickness was determined on hematoxylin and eosin (H&E)-stained bladder sections following previously described procedures [32]. A quantitative analysis was performed using a Leica Qwin 500 Image Analyzer (Leica Imaging Systems Ltd., Cambridge, UK). Measurements were obtained at a magnification of ×10 and expressed in micrometers (µm). For each animal, 15 measurements were taken from five separate tissue sections, and the mean value for each rat was used for the statistical analysis. The results represent the average urothelial thickness across animals within each experimental group.

### 4.9. Determining the Expression Levels of cFos in Central Micturition Areas

The expression of the immediate early gene c-Fos was evaluated in key brain regions associated with micturition control. Using the stereotaxic atlas of the rat brain, the pontine micturition center (PMC), ventrolateral periaqueductal gray (vlPAG), and medial preoptic area (MPA) were identified with reference to the bregma. Approximately ten coronal sections per brain region were collected from each rat. The analyzed coordinates were defined as follows: PMC, bregma −9.68 to −9.80 mm; vlPAG, bregma −7.64 to −8.00 mm; and MPA, bregma −0.26 to 0.80 mm [52]. A quantitative analysis of c-Fos immunoreactivity within these regions was subsequently performed.

### 4.10. Statistical Analysis

All quantitative data were analyzed using a two-way analysis of variance (ANOVA) followed by Tukey’s post hoc test for multiple comparisons (Statistica v.10, StatSoft Inc., Tulsa, OK, USA). The results are presented as the mean ± standard error of the mean (SEM). Differences were considered statistically significant at *p* < 0.05, corresponding to a 95% confidence level.

* or ^ *p* < 0.05; ** or ^^ *p* < 0.01; *** or ^^^ *p* < 0.001, **** or ^^^^ *p* < 0.0001;

* Significantly different from the CON group;

^ Significantly different from the CYP group.

## 5. Conclusions

In summary, SPC robustly mitigated cyclophosphamide (CYP)-induced cystometric dysfunction and biochemical alterations characteristic of iatrogenic hemorrhagic cystitis. Its protective effects appear to be mediated through coordinated modulation of proinflammatory cytokines, neurotransmitter signaling, and intercellular signal transduction. These findings position SPC as a compelling therapeutic candidate and warrant further translational investigation for the management of CYP-induced bladder injury.

## Figures and Tables

**Table 1:** The effects of SPC on cyclophosphamide (CYP)-induced changes in cystometric parameters.

	Control	CYP	SPC	CYP + SPC
Filling phase				
Basal pressure, BP (cm H_2_O)	2.5 ± 0.79	5.1 ± 0.99 ****	2.5 ± 0.62 ^^^^	3.2 ± 0.91 ^^^^
Threshold pressure, TP (cm H_2_O)	7.2 ± 2.0	4.5 ± 1.1 ***	6.6 ± 1.5 ^^^	7.8 ± 2.1 ^^^^
Detrusor overactivity index, DOI (cm H_2_O/mL)	40 ± 17	515 ± 317 ****	39 ± 25 ^^^^	213 ± 132 ^^^^
Non-voiding contraction frequency, FNVC (times/filling phase)	0.3 ± 0.15	5.6 ± 1.4 ****	0.28 ± 0.11 ^^^^	1.2 ± 0.7 ^^^^
Volume threshold to elicit NVC, VTNVC (%)	66 ± 9.8	39 ± 16 ****	55 ± 17 ^	64 ± 16 ^^^
Non-voiding contraction amplitude, ANVC (cm H_2_O)	2.1 ± 0.16	6.3 ± 1.1 ****	2.3 ± 0.45 ^^^^	3.2 ± 0.64 ^^^^
Bladder compliance, BC (mL/cm H_2_O)	0.29 ± 0.079	0.16 ± 0.036 ****	0.24 ± 0.061 ^^	0.22 ± 0.063 ^
Voiding phase				
Micturition voiding pressure, MVP (cm H_2_O)	46 ± 7.5	42 ± 12	49 ± 13	45 ± 14
Intercontraction interval, ICI (s)	1117 ± 17	672 ± 182 ****	994 ± 133 ^^^^	946 ± 193 ^^^
Voided volume, VV (mL)	1 ± 0.16	0.47 ± 0.19 ****	0.97 ± 0.31 ^^^^	0.78 ± 0.27 ^^
Post-void residual, PVR (mL)	0.062 ± 0.02	0.055 ± 0.017	0.074 ± 0.016 ^	0.068 ± 0.019
Area under the pressure curve, AUC (cm H_2_O/s)	13 ± 2.5	24 ± 6.8 ****	13 ± 2.9 ^^^^	16 ± 3.7 ^^^^

Values are expressed as the mean ± SD. ^ *p* < 0.05; ^^ *p* < 0.01; *** or ^^^ *p* < 0.001, **** or ^^^^ *p* < 0.0001. * significantly different from the control group. ^ significantly different from the CYP group.

## Data Availability

The data presented in this study are available from the corresponding authors upon request.

## Abbreviations

The following abbreviations are used in this manuscript:

ANVC	non-voiding contraction amplitude (cm H_2_O)
AUC	area under the pressure curve (cm H_2_O/s)
BC	bladder compliance (mL/cm H_2_O)
BDNF	brain-derived neurotrophic factor (pg/mL)
BP	basal pressure (cm H_2_O)
CGRP	calcitonin gene-related peptide (pg/mL)
DO	detrusor overactivity
DOI	detrusor overactivity index (cm H_2_O/mL)
FNVC	non-voiding contraction frequency (times/filling phase)
HB-EGF	heparin-binding EGF-like growth factor (pg/mL)
HPX	hemopexin (pg/mL)
ICI	intercontraction interval (s)
IGFBP-3	insulin-like growth factor-binding protein 3 (pg/mL)
IL-1β	interleukin 1-β (pg/mL)
IL-6	interleukin 6 (pg/mL)
MAL	malondialdehyde (pg/mL)
MVP	micturition voiding pressure (cm H_2_O)
NGF	nerve growth factor (pg/mL)
NIT	3-nitrotyrosine (pg/mL)
OAB	overactive bladder syndrome
OCC	occludin (pg/mL)
OCT3	organic cation transporter 3 (pg/mL)
ORM1	orosomucoid-1 (pg/mL)
PAC1	pituitary adenylate cyclase receptor 1 (pg/mL)
PVR	post-void residual (mL)
ROCK1	Rho kinase (pg/mL)
SNAP-23	synaptosome-associated protein 23 (pg/mL)
SNAP-25	synaptosome-associated protein 25 (pg/mL)
SV2A	rat synaptic vesicle glycoprotein 2A (pg/mL)
T-H	Tamm-Horsfall protein (pg/mL)
TNF-	tumor necrosis factor alpha (pg/mL)
TP	threshold pressure (cm H_2_O)
UT	urothelium thickness (mikrom)
VAChT	vesicular acetylcholine transporter (pg/mL)
VTNVC	volume threshold to elicit NVC (%)
VV	voided volume (mL)
Z01	tight junction protein 1 (pg/mL)
